# Talking About Life Experiences: Protocol for Treatment Development and a Feasibility Trial of a Novel Narrative Discourse Intervention for Individuals With Traumatic Brain Injury

**DOI:** 10.2196/86329

**Published:** 2026-04-24

**Authors:** Karen Le, Carl Coelho, Richard Feinn, Joanna M Fiszdon

**Affiliations:** 1 Audiology and Speech Pathology Service VA Connecticut Healthcare System West Haven, CT United States; 2 Department of Neurology School of Medicine Yale University New Haven, CT United States; 3 Research Service VA Connecticut Healthcare System West Haven, CT United States; 4 Frank H Netter MD School of Medicine Quinnipiac University North Haven, CT United States; 5 Psychology Service VA Connecticut Healthcare System West Haven, CT United States; 6 Department of Psychiatry School of Medicine Yale University New Haven, CT United States

**Keywords:** acquired brain injury, communication, communication disorders, discourse, intervention, narrative, rehabilitation, traumatic brain injury, treatment, Veterans

## Abstract

**Background:**

Cognitive-communication disorders are pervasive following traumatic brain injury (TBI), disrupting communication at the level of discourse and social interaction. Discourse impairments impact functioning across major life domains, such as work and social relationships, and overall quality of life. Problems with discourse affect all severity levels of TBI and persist over time. Veterans may experience even greater functional decline due to comorbid health conditions (eg, posttraumatic stress disorder, pain). The functional impact and chronicity of discourse impairments following TBI underscore the importance of treating these impairments.

**Objective:**

This study aims to (1) develop, refine, and manualize a treatment protocol targeting narrative discourse—Talking About Life Experiences (TALE)—in people with TBI and (2) evaluate the feasibility of the TALE discourse treatment in a pilot randomized controlled trial (RCT).

**Methods:**

This is a stage 1a (phase 1) and stage 1b (phase 2) treatment development study aligned with the Stage Model of Behavioral Therapies Research. All participants will be individuals with TBI and communication difficulties. In the treatment development phase (phase 1), 5-10 participants will be recruited to obtain key stakeholder feedback for treatment refinement. In the feasibility pilot phase (phase 2), 40 participants with TBI and communication difficulty will be randomized to receive the TALE intervention or treatment as usual. We will obtain information on the tolerability, acceptability, and feasibility of recruitment for the TALE intervention, as well as preliminary data on treatment delivery, assessment methods, and treatment effects. Assessments will be conducted at baseline, posttreatment, and at 1-month follow-up and will include measures of discourse ability, cognition, mental health, pain, functional communication, and daily functioning. Feedback regarding assessment and treatment will also be collected via surveys and exit interviews.

**Results:**

This study was funded in October 2021. Recruitment for phase 1 began on September 26, 2022. Seven participants were enrolled. Phase 1 concluded on November 17, 2023. Recruitment for phase 2 began on November 27, 2023. Thirty-four participants were enrolled. Data collection was completed on November 3, 2025. Data analysis has been conducted concurrently with data collection and is expected to continue until early-mid 2026. Results are expected to be published in peer-reviewed journals in late 2026 and early 2027.

**Conclusions:**

Stakeholder feedback from the phase 1 treatment development trial will facilitate refinement of the TALE protocol in preparation for the phase 2 feasibility pilot RCT. Results from phase 2 will be used to determine the tolerability, acceptability, and feasibility of methods. Findings from this study will inform the development and implementation of a future fully powered RCT evaluating treatment efficacy.

**Trial Registration:**

ClinicalTrials.gov NCT05008419; https://clinicaltrials.gov/ct2/show/NCT05008419

**International Registered Report Identifier (IRRID):**

DERR1-10.2196/86329

## Introduction

Traumatic brain injury (TBI) is a substantial public health problem in the United States [1], and 1%-2% of the civilian US population has a long-term disability due to TBI. Among Service Members and Veterans (SMV), the rates of TBI are much higher, affecting an estimated 20% of the population [2,3]. Cognitive-communication disorders are widespread in TBI, with incidence estimates as high as 80%-100% [4]. The hallmark of cognitive-communication disorders in TBI is discourse impairment, which significantly impacts everyday functioning [5-7] and has been observed at all levels of TBI severity [7-9]. The functional impact and pervasiveness across severity levels render discourse impairments an important treatment target. To date, little work has been done to develop treatments for discourse impairments.

Discourse is language beyond the level of single sentences that conveys meaning and comprises much of daily communication in natural settings, such as the home, school, or workplace. Discourse skills are crucial for successful communication and functioning in these environments. Conversely, social integration, occupational functioning, and quality of life are negatively impacted by discourse impairments following TBI [10,11]. Furthermore, discourse impairments are persistent and often do not resolve over time [12,13]. Poor discourse skills affect return to work and the ability to sustain meaningful relationships, which can lead to loneliness and depression [14,15].

Although TBI is a known disruptor of discourse ability, additional factors, such as psychiatric or neurologic comorbidities, may also influence discourse production and exacerbate discourse impairments. For example, narratives produced by some individuals with posttraumatic stress disorder (PTSD) have been found to differ in the use of pronouns, death-related words, cognitive words, sensory/perceptual references, and temporal concepts [16]. Posttraumatic headache severity has been associated with greater pauses and slower speech, which may reflect disruption not only to speech motor control but also to cognitive-linguistic processes underlying discourse production [17]. Mental health (MH) conditions, such as PTSD, anxiety, and depression, and neurologic conditions, such as pain and headache, often co-occur with TBI in SMVs [18,19]. Thus, discourse impairments in SMVs with TBI are likely compounded by physical health and MH comorbidities, further impairing functioning [18,20]. Remediating discourse impairments in Veterans with TBI would enhance communication, providing a gateway to increased participation across a number of life domains and improved functioning within these domains.

Currently, discourse treatment protocols are scarce and represent a nascent area of research [21]. Efforts to improve social communication have focused primarily on training social skills or the communication partner rather than targeting the impaired discourse of the individual with TBI [21-23]. Generalization of social skills training to improvements in narrative discourse ability, an important component of daily communication [24], has not been examined. The few attempts to improve narrative discourse ability in TBI have had very small sample sizes of 1-10 participants and have focused on improving discourse content or organization separately, resulting in small treatment effects and limited carryover [25-30]. To date, no manualized treatments exist that specifically target discourse in people with TBI, and no studies have integrated treatment approaches by targeting both discourse content and organization in a social communication context. An integrated and manualized treatment approach would directly treat discourse impairments and maximize the potential for functional generalization to social interactions.

**Figure 1:**
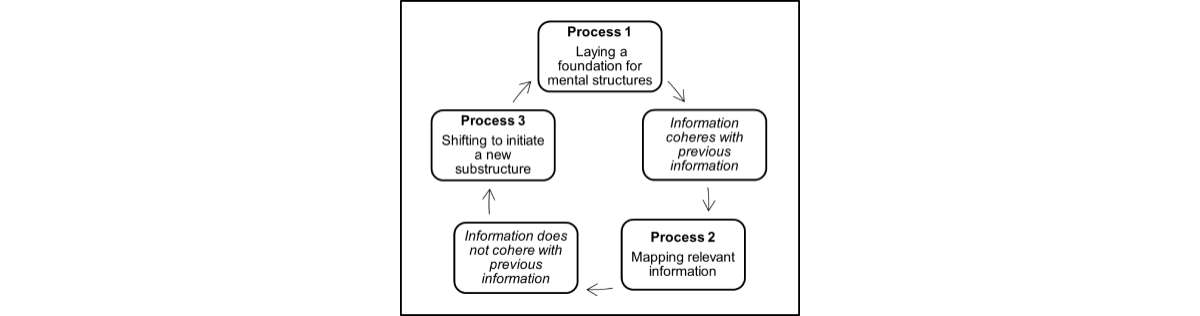
Structure Building Framework.

The Structure Building Framework (SBF), a discourse comprehension model, has shown potential to guide the development of discourse treatments [31]. The SBF is a cognitive discourse processing model that stipulates that comprehension of discourse involves the construction of mental representations (ie, structures) through 3 key building processes: (1) laying a foundation; (2) mapping relevant information onto that foundation; and (3) shifting, as needed, to initiate a new substructure when incoming information is unrelated to the currently activated substructure (Figure 1). Two general mechanisms operate in building mental structures: the enhancement of relevant information and the suppression of irrelevant information. While the SBF was developed to explain discourse comprehension, the same mechanisms are purportedly involved in discourse production [32], and the SBF is the only cognitive model that has been applied to explain both discourse comprehension and production in neurologic and psychiatric populations [32-34]. The SBF has been useful in explaining discourse impairments in TBI and conceptualizes both discourse outcomes and the processes that form discourse [35,36]. As such, the SBF provides an excellent blueprint to guide treatment development. For the proposed study, the SBF will be used to guide remediation of both story content and story organization abilities, as it provides an organizational framework for mapping story information.

The overarching goal of the proposed study is to advance cognitive-communicative intervention for people with TBI by developing and evaluating a manualized, evidence- and theoretically based narrative discourse treatment that addresses breakdowns in both story content and organization. Specific aims are as follows:

- Aim 1: Develop, refine, and manualize a narrative discourse treatment targeting story content (completeness, accuracy, and coherence) and story organization (story grammar knowledge and episode formation).
- Aim 2: Design and conduct a pilot study to assess the feasibility and tolerability of the treatment and to evaluate methods for a larger-scale study.

The study will have 2 phases, aligned with the specific aims. Phase 1 will focus on treatment development, while phase 2 will evaluate treatment feasibility. During phase 1, 5-10 participants will be recruited to obtain key stakeholder feedback for treatment refinement. In phase 2, 40 participants with TBI and communication difficulty will be randomized to receive the Talking About Life Experiences (TALE) intervention or treatment as usual (TAU). We will obtain information on the tolerability, acceptability, and recruitment feasibility of the TALE intervention, as well as preliminary data on treatment delivery, assessment methods, and treatment effects.

## Methods

### Protocol Version and Access

This protocol is version 11.0 (10/22/2025) of the ClinicalTrials.gov-registered protocol, which replaces version 10.0 (11/20/2024). Revisions were made to update the recruitment status, study status, masking status, enrollment number, and contact information at the request of the funding agency. The protocol is summarized in the trial registry. The protocol and statistical analysis plan are described in detail in this manuscript.

### Study Design

**Figure 2:**
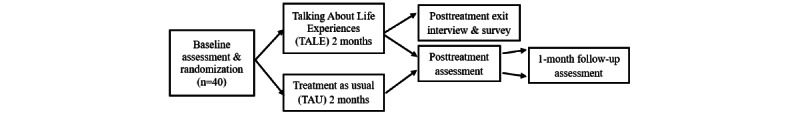
Feasibility RCT study flow.

The proposed research project is an early-stage treatment development study focused on the conceptualization and feasibility evaluation of a narrative discourse treatment for participants with TBI. The project will consist of (1) initial treatment and manual development and refinement with a small participant sample (n=5-10; aim 1; phase 1: treatment development) and (2) a treatment feasibility trial in a separate sample of participants (n=40) to obtain information regarding the tolerability and acceptability of the proposed discourse treatment, as well as preliminary data on treatment delivery and assessment methods and preliminary information about treatment effects (aim 2; phase 2: feasibility trial). The feasibility trial will be a 2-arm (TALE intervention vs TAU), parallel-group randomized controlled trial (RCT) with an allocation ratio of 1:1. All participants will undergo screening. Participants in the phase 2 feasibility trial will complete comprehensive assessments at baseline, end of treatment (following approximately 14-18 sessions over 2-3 months), and at 1-month follow-up (Figure 2). Treatment tolerability and acceptability data will be collected in both phases 1 and 2. See Table 1 for assessment timelines and a summary of instruments.

**Table 1:** Timeline of measures and summary of instrument psychometrics.

Instrument/measure	Duration	Measurement domain	Psychometrics	Screening	Baseline	After treatment	1-month follow-up
Psychosocial intake	10 minutes	Demographic and psychosocial	N/A^a^	✓	N/A	N/A	N/A
Ohio State University Traumatic Brain Injury Identification	5 minutes	Lifetime history of traumatic brain injury	High interrater reliability and good predictive validity with traumatic brain injury factors (eg, injury age, persistent symptoms) [37]	✓	N/A	N/A	N/A
4-item Mayo-Portland Adaptability Inventory	10 minutes	Daily functioning and life participation	High person^b^ and item reliability; as well as good concurrent and predictive validity with disability, functional, and cognitive measures [38]	✓	N/A	✓	✓
Rancho Los Amigos Level of Cognitive Functioning Scale—Revised	1 minute	Functional cognitive impairment	High interrater and test-retest reliability; as well as good concurrent and predictive validity with functional outcome and cognitive measures [39]	✓	N/A	N/A	N/A
Bedside Western Aphasia Battery—Revised	15 minutes	Aphasia, language fundamentals, and auditory processing	High interrater and test-retest reliability; as well as convergent, discriminant, and concurrent validity with language and communication measures [40,41]	✓	N/A	N/A	N/A
Discourse Comprehension Test	30 minutes	Discourse comprehension	High test-retest reliability and strong correlations with auditory comprehension and communicative processing measures [42,43]	N/A	✓	✓	✓
Story completeness	40 minutes (discourse sample collection concurrent with all discourse production measures)	Discourse production (content)	High interrater reliability and moderate correlations with working and declarative memory and executive function measures [35,44]	N/A	✓	✓	✓
Global coherence	40 minutes (discourse sample collection concurrent with all discourse production measures)	Discourse production (content)	High interrater reliability and small to moderate correlations with attention, working, and episodic memory measures [45,46]	N/A	✓	✓	✓
Story grammar	40 minutes (discourse sample collection concurrent with all discourse production measures)	Discourse production (organization)	High interrater reliability and moderate correlations with declarative memory and executive function measures [35,44,47]	N/A	✓	✓	✓
Story Goodness Index	40 minutes (discourse sample collection concurrent with all discourse production measures)	Discourse production (overall quality)	Same as story completeness and story grammar (Story Goodness Index based on these measures)	N/A	✓	✓	✓
Wechsler Adult Intelligence Scale—Fourth Edition Digit Span	15 minutes	Cognition	High interrater reliability and high concurrent validity with other cognitive measures [48]	N/A	✓	✓	✓
Wechsler Memory Scale—Fourth Edition Logical Memory 1	5 minutes	Cognition	High test-retest reliability and strong construct validity [49,50]	N/A	✓	✓	✓
Repeatable Battery for the Assessment of Neuropsychological Status Animal Fluency	1 minute	Cognition	High internal consistency and strong concurrent validity [51]	N/A	✓	✓	✓
Delis-Kaplan Executive Function System Tower Test	30 minutes	Cognition	High discriminant validity and moderate to high ecological validity [52,53]	N/A	✓	✓	✓
Traumatic Brain Injury Quality of Life Communication Item Bank	3 minutes	Functional communication	High internal consistency and good convergent and discriminant validity with related functional communication measures [54]	N/A	✓	✓	✓
Posttraumatic Stress Disorder Checklist for DSM-5^c^	5 minutes	Posttraumatic stress disorder	High test-retest reliability and excellent convergent and discriminant validity [55]	N/A	✓	✓	✓
7-item Generalized Anxiety Disorder Scale	5 minutes	Anxiety	High test-retest reliability and good-excellent criterion and convergent validity [56]	N/A	✓	✓	✓
9-item Patient Health Questionnaire	2 minutes	Depression	High test-retest reliability and good construct and criterion validity [57]	N/A	✓	✓	✓
Pain, Enjoyment of Life and General Activity Scale	1 minute	Pain	High sensitivity and clinical validity [58]	N/A	✓	✓	✓
6-item Headache Impact Test	2 minutes	Headache	High internal consistency, test-retest reliability, and discriminant validity [59]	N/A	✓	✓	✓

^a^N/A: not applicable.

^b^Person reliability refers to the consistency of an individual’s performance on a measure over time.

^c^DSM-5: Diagnostic and Statistical Manual of Mental Disorders, Fifth Edition.

### Participants

Participants for both phases 1 and 2 will be Veterans with TBI who report communication difficulty that interferes with activities of daily living. Inclusion criteria will be as follows: at least 3 months after injury (ie, not in the acute TBI stage); no penetrating head injury per chart review and self-report; adequate orientation and ability to follow directions, as determined by the ability to answer intake questions and performance on the Bedside Western Aphasia Battery—Revised (WAB-R) [40]; English language fluency; adequate hearing and visual acuity; age 18 years or older; a minimum rating of VII (automatic, appropriate) on the Rancho Los Amigos Level of Cognitive Functioning Scale—Revised (LCFS) [60]; willingness and ability to participate in telehealth sessions; and stable housing with a safe, private, and quiet environment at home to engage in telehealth sessions.

Exclusion criteria will be as follows: psychotic disorder; neurological illness (eg, stroke, dementia, Parkinson disease); aphasia or auditory processing disorder; and current (approximately past 30 days) problematic alcohol or substance use, as judged by the principal investigator (PI; KL). An exception will be made for marijuana, as a number of Veterans use it occasionally to manage certain ailments; however, marijuana use must not interfere with daily functioning.

Participants will be asked to identify a significant other (eg, spouse, family member, or friend) who is able and willing to serve as an informant to verify discourse ability before and after treatment. If a participant does not have a significant other or does not wish to identify one to serve as an informant, the participant may still enroll in the study. Individuals engaged in MH treatment for concomitant conditions (eg, PTSD, anxiety, depression) will not be excluded.

### Study Procedures

#### Overview

All study procedures will take place at or through the VA Connecticut Healthcare System (VACHS) in West Haven, Connecticut. All telehealth delivery will be administered using VACHS-approved platforms.

Participants will be recruited from VACHS through the Polytrauma Support Clinic Team, Speech Pathology Service, and flyers. The study team will access Polytrauma/TBI and Speech Pathology consult lists, as well as patient lists generated for biweekly Polytrauma/TBI team meetings, and will conduct chart reviews of patients on these lists. The study team will ask the aforementioned recruitment sources to describe the study to potentially eligible participants identified by the study team or the recruitment sources and to ask whether they would be interested in learning more about the study. If so, their names and contact information will be forwarded to the study team.

Following informed consent and screening, phase 1 participants will undergo a preliminary version of the TALE treatment. No baseline, posttreatment, or follow-up assessments will be administered during this initial treatment and manual development phase, as the primary focus at this stage is to refine the intervention in preparation for the subsequent treatment feasibility trial. Administration of the treatment will occur serially to allow for iterative revision and refinement. Thus, in the phase 1 treatment development trial, feedback from each participant regarding their experience with TALE will be used to revise the treatment for the next participant.

#### Phase 1 and Phase 2 Screening

Individuals who express interest in the study will be prescreened by telephone to confirm that they meet major eligibility criteria. Those deemed eligible will be invited for an in-person initial screening.

In addition to reviewing the medical record, screening measures will be used to verify eligibility for participation during the in-person screening (Table 1). A psychosocial intake will be administered to collect demographic and psychosocial data. Screening measures will include the Ohio State University Traumatic Brain Injury Identification [37], the 4-item Mayo-Portland Adaptability Inventory (MPAI-4) [61], the LCFS [60], and the Bedside WAB-R (cutoff score: ≥ 93 for inclusion) [40]. The in-person screening is expected to take approximately 1 hour.

#### Phase 2 Baseline, Posttreatment, and Follow-Up Testing

Baseline and posttreatment assessments will take approximately 2.5 hours for each assessment period. The first posttreatment assessment will occur after treatment ends, approximately 3 months after the first treatment session. The follow-up assessment will occur about 1 month thereafter. The posttreatment and follow-up assessments will be the same as those administered at baseline. As the MPAI-4 is already being administered at screening, it will be repeated only after treatment and at the 1-month follow-up. Given that baseline testing will occur within about 1 month of screening and that all participants will be beyond the acute stage of TBI, when most spontaneous recovery is expected to occur, MPAI-4 performance is expected to be stable during the period between screening and baseline. This approach reduces participant burden associated with readministration of the same assessment within a short interval. Informants will complete the TBI Quality of Life Communication Item Bank (TBI-QOL COM) short form [54] to provide collateral information about functional communication. Participants will be offered breaks and, if requested, testing will be split into multiple sessions. To accommodate participants and allow for testing flexibility, self-report surveys and several assessments may also be administered by phone or virtually to shorten the in-person testing session. The modality of assessments will be tracked.

#### Treatment

In both phases 1 and 2, participants will receive treatment from a trained study therapist. Individual, hour-long sessions will occur 2 times a week over approximately 2-3 months. In the phase 1 treatment development trial, there will be about 16-20 sessions in total. Feedback obtained in phase 1 will be used to reduce the number of sessions to about 16. Thus, following the iterative revision process, we anticipate that the number of sessions will range from 14 to 18 for the phase 2 feasibility trial.

Participants will be offered the choice of telehealth or in-person sessions and will be encouraged to adhere to their chosen treatment delivery format for the duration of treatment. Some flexibility will be allowed to accommodate participants who wish or need to change formats during treatment. Although the treatment structure aims to deliver 2 sessions each week, some flexibility will be allowed to accommodate issues such as missed appointments, holidays, leave, and “catch-up” appointments, which may result in 3-4 sessions in a week. Some participants may not be able to complete “catch-up” sessions. As such, to allow flexibility, study participation may extend up to 6 months.

#### Feedback Questionnaires and Exit Interview

Treatment questionnaires will be administered to all phase 1 participants and to phase 2 participants randomized to the TALE intervention arm at the end of treatment to ascertain information regarding training satisfaction and training content. Assessment questionnaires will be administered to all phase 2 participants at the posttreatment and follow-up assessments. The questionnaires will take approximately 15 minutes. A 30- to 45-minute semistructured exit interview will also be administered posttreatment to obtain qualitative feedback regarding the treatment. The questionnaires and exit interview are further described in the “Primary Outcome Measures: Treatment Feasibility” section.

#### Randomization and Blinding

The individuals (n=5-10) recruited in phase 1 will not be randomized, as phase 1 focuses on the initial treatment and manual development phase. For phase 2, individuals will be randomized to either the discourse treatment condition, TALE, or a TAU condition using permuted block randomization (Figure 2). Randomization will be stratified by TBI severity (mild vs nonmild) and education (high school vs college degree). Grading of injury severity will follow VA/Department of Defense TBI severity guidelines [62]. The study statistician (RF) will generate the sequentially numbered randomization scheme. The PI will assign participants according to the randomization scheme. The randomization sequence will be concealed from the study assessors and study therapist, except for the PI, who will also conduct assessments. Personnel who enroll and assign participants will be separate staff. Personnel who enroll participants will not have access to the randomization scheme.

This is an unblinded feasibility study. Participants and outcome assessors will not be blinded to intervention assignments. Blinding was considered but is not feasible for practical reasons, namely, personnel constraints. The research team does not currently have sufficient staff to carry out the study while maintaining blinding. Given that the primary outcomes focus on feasibility rather than treatment effects, the risk of bias due to lack of blinding was deemed to be low. Blinding will be implemented in future fully powered trials of the intervention, in which hypothesis testing (eg, examining treatment efficacy) will be possible.

### Treatment Conditions

#### Overview of the TALE Experimental Treatment

TALE aims to improve narrative discourse ability by targeting story organization and story content in the context of social communication. Story organization will be addressed through story grammar training by teaching specific episode elements: setting, initiating event, attempt, and direct consequence. Lay language will be used to convey these elements (ie, setting, trigger, action, and result), and the acronym “STAR” will be implemented as a central concept and mnemonic device to support learning of these elements. Story content will be addressed by targeting aspects foundational to story completeness and global coherence. Thus, treatment of story content will encompass lessons on main ideas and details, intentions, and word retrieval.

The manual will feature individual session content along with clinical examples, scripts, and vignettes, as well as handouts and homework assignments. Treatment materials will include publicly available photos, videos, and other media to elicit narratives. Microsoft PowerPoint will be used to deliver lessons, allowing for the presentation of mixed media.

All handouts and homework assignments for each week will be provided in paper form, electronically, or both, depending on the participant’s preference. Although participants will be given about 1 week to complete homework assignments, homework will be assigned at each session to address aspects not yet completed or to provide continued practice for completed components. Homework will include learning and practicing strategies and activities to promote functional carryover of skills learned in session to interactions with the informant and other communication partners in the participant’s daily environments.

#### TALE Treatment Development

The initial discourse training stimuli will be developed by the first author (KL) and distributed to the second (CC) and last authors (JMF) for feedback on the following training elements: organization, content, appropriateness of training techniques, and appropriateness of training stimuli. The treatment development process will be iterative and will occur on a monthly basis during the initial development phase. The expected outcome of this phase will be a preliminary version of the treatment manual, specifying session length, structure, the specific training exercises to be used in each session, and how training should progress through the activities.

A panel of 4 experts and stakeholders has been recruited to provide an external review of the treatment protocol and materials during treatment development. We will also recruit a Veteran with TBI, who will not be a participant in the study, to serve as a key stakeholder reviewer.

A preliminary version of the treatment will be administered serially to 5-10 participants (phase 1: treatment development). Feedback from these participants will be obtained at the end of treatment using the measures (training satisfaction and training content questionnaires) described in the “Primary Outcome Measures: Treatment Feasibility” section. Brief, informal feedback measures will also be obtained after each session.

Using information obtained during evaluation of the preliminary version of the discourse treatment protocol, we will continue to revise training content and refine manual development. Treatment refinement, based on information obtained from the serial administrations, will continue until the PI and study team concur that information from administered sessions, including but not limited to participant feedback, has been appropriately incorporated into the manual and no further modifications are warranted. This final stage of treatment development will culminate in a near-final version of the treatment manual and training materials in preparation for use in the subsequent treatment feasibility trial (phase 2).

#### TALE General Treatment Approach

The proposed discourse treatment approach uses systematic instruction as a foundation to guide learning [63]. Systematic instruction is an evidence-based approach to optimize learning in individuals with learning impairments through structured training that incorporates (1) explicit models, (2) minimization of errors during the initial learning phase, (3) strategies to promote learner engagement, and (4) carefully guided practice to promote mastery, maintenance, and generalization. Treatment techniques that have the potential to improve discourse ability will be implemented, including feedback; use of simulated and actual social contexts; functional practice of learned skills; development of metacognitive and metalinguistic strategies; and hierarchical training [21].

General therapeutic techniques will be used throughout the phases of treatment, including brief and simple instructions, visual cues/reminders, and “teach-back” repetitions/summaries of information by the participant to check understanding. Scaffolding and feedback will be provided to correct mistakes and guide participants toward target productions. Training will be incremental and will build upon each earlier section of treatment. The discourse treatment will also employ general learning principles, including errorless learning and spaced retrieval. The treatment will build upon prior studies by integrating the aforementioned techniques and components into a systematic instruction approach to maximize the potential for change.

There will be 3 treatment sections (discussed later). Treatment components specific to each section are described in the following sections.

#### TALE-Specific Treatment Components, as Informed by the SBF

The SBF will be used to guide remediation of both story content and story organization abilities, as it provides an organizational framework for mapping story information. Participants will be taught to lay the foundation for a story through instruction in the story grammar elements that form episodes. They will then learn how to build upon those elements as they produce the story episode (eg, mapping relevant information), which will involve judgments of the relevance and salience of story content. When the story topic changes, participants will be taught to recognize the transition and shift to form new episodes to support the novel story content. Throughout the treatment process, participants will actively develop and hone the ability to enhance or suppress story information depending on its relevance and salience (see Textboxes 1-3).

**Part 1: Overview and general communication strategies.**

Trainees will receive education about stories, including what a story is, the elements of a story, and what makes a good story. Story content and story organization will be reviewed as 2 important dimensions of effective stories. The process of formulating stories, based on the Structure Building Framework, will be introduced. Trainees will listen to and review transcripts of examples of both well-formed and incomplete, disorganized stories and will be asked to identify differences between these types of stories. Trainees will also learn about communication, self-advocacy, and word-finding strategies. At the end of this section, trainees should be able to teach back major aspects of story content and organization, communication, and word-finding strategies, and understand what makes a complete, organized story.

**Part II: Story organization.**

Training will focus on story organization, using the STAR (ie, setting, trigger, action, and result) acronym as a central concept. Story organization will be trained before story content, as it forms the story’s foundation, which is consistent with the Structure Building Framework. Trainees will produce stories using a variety of stimuli, such as picture scenes and sequences, and video clips. Trainees will be guided in self-reflection on their story transcripts using structured training prompts. Trainees will identify story grammar elements that are present or absent in their stories and learn to lay the foundation of their stories using these elements. At the end of this section, participants should be able to produce well-organized stories, as evidenced by the presence of all story grammar elements.

**Part III: Story content and integration.**

Training will focus on story content, including word selection, intentions, and story informativeness and completeness. Trainees will learn word retrieval strategies and apply them to structured and unstructured stories. Trainees will learn about communicative intentions and practice conveying various intentions in stories. For training in story informativeness and completeness, participants will again produce stories using a variety of stimuli, which may be recorded and transcribed to facilitate self-reflection. This section will incorporate teaching of the Structure Building Framework’s mapping and shifting processes and will integrate this information with story organization strategies learned previously. Trainees will learn to evaluate the relevance and salience of information and the difference between a main idea and a detail to tell stories that meet the information needs of the situation. At the end of this section, trainees should be able to produce informative, complete, and organized stories, as evidenced by meeting a threshold criterion (eg, 80%) for main ideas and details and story grammar elements.

#### Overview of the TAU Comparison Condition

Veterans randomized to TAU will continue to receive standard rehabilitative care if they choose to engage in these services. This may include other cognitive-communication treatment, speech-language therapy, psychosocial treatment, physical therapy, occupational therapy, pain management, and pharmacological interventions. No TAU services specifically provide discourse treatment.

### Measures

#### Primary Outcome Measures: Treatment Feasibility

##### Tolerability

For phase 1 (treatment development) and phase 2 (feasibility trial), treatment tolerability will be assessed by measuring session attendance and study retention (percentage of withdrawals and percentage participating in each assessment battery). Information will also be obtained on completion of homework assignments.

##### Acceptability

Treatment acceptability will be assessed in phase 1 and phase 2 through questionnaires administered to study participants at the end of treatment to obtain information about training satisfaction and content. Regarding training satisfaction, participants will be asked how useful the training was in helping them produce stories, how much they learned as a result of the training, and whether they would recommend the training to others. Feedback will also be obtained regarding satisfaction with session duration, session frequency, overall treatment length, and in-person versus telehealth delivery, as well as assessment duration, frequency of assessments, and assessment delivery format. Responses will be on a 5-point Likert scale.

Questionnaires to obtain feedback about training content will address each training component. Participants will be asked how helpful they felt the training was, whether the training progressed at a suitable rate for them, and whether they felt that the training would be useful in future storytelling situations. Responses will be on a 5-point Likert scale. Participants will also be asked about the components of the training they found helpful in formulating stories. The surveys will take approximately 15 minutes.

For both treatment development phases, a 30-45-minute semistructured exit interview will be administered after treatment to obtain qualitative feedback regarding the treatment, including reflections on the participant’s experience of the discourse treatment, suggestions for improving the treatment, preferences for treatment delivery format and duration, barriers and facilitators to treatment engagement, and feedback on specific treatment components.

##### Recruitment and Study Flow

For phase 2, we will obtain pilot data on recruitment and study flow. The following data will be collected: number of calls received about the study, number of potential participants who pass the brief telephone screening and sign informed consent, number of participants eligible following the study intake screening, and number randomized. All data necessary for a CONSORT (Consolidated Standards of Reporting Trials) flowchart will be obtained. This study follows the SPIRIT (Standard Protocol Items: Recommendations for Interventional Trials; Multimedia Appendix 1; also see Multimedia Appendix 2 for reporting of essential clinical trial elements.

#### Screening Measures

This section describes the 5 screening measures mentioned in the study procedures. The intake will collect psychosocial and demographic data. Major intake sections include housing, employment, current medications, rehabilitation history, MH and neurologic history, and substance use. The Ohio State University Traumatic Brain Injury Identification is a standardized screening tool for assessing the lifetime history of TBI [37]. The MPAI-4 is frequently used in the VA to assess impairment and functioning following TBI and will be used as a key screening tool for the proposed study [61]. The MPAI-4 items related to verbal communication, alcohol use, and drug use will be used to ensure participants meet the inclusion criteria on those items. The LCFS will provide a measure of the severity of current functional cognitive impairment in carrying out activities of daily living, as well as an index of orientation and ability to follow directions [60]. The Bedside WAB-R will be used to rule out the presence of aphasia and auditory processing problems (cutoff score: ≥93 for inclusion) [40]. To pass the Bedside WAB-R, individuals must pass the 3 subsections that assess auditory-based skills. See Table 1 for a summary of screening measures and corresponding psychometrics.

#### Assessment Battery Measures

##### Assessment Framework and Outcome Domains

Assessment will comprise measures of discourse ability, functional communication, daily functioning, cognition, MH, pain, and headache. The assessment battery was developed based on MacDonald’s model of cognitive-communicative competence, which provides guidance for evidence-based cognitive-communication interventions after TBI [64]. The 7 interconnected domains of the model that should be considered for treatment are the individual, context/environment, cognition, communication, physical/sensory functioning, and emotional/psychosocial functioning, all of which contribute to the final domain, communication competence. The proximal outcomes will be the discourse measures, reflecting the communication construct. Given the functional impact of discourse impairments following TBI, we expect that there will also be changes in functional communication and daily functioning, reflecting the constructs of the individual, environment, and communication competence. Given the interconnectedness among domains, cognitive, neurologic, and MH factors will also be examined, as they may influence discourse performance and may change as a result of treatment. See Table 1 for the timeline of assessments and a summary of instrument psychometrics.

##### Discourse Ability

Discourse comprehension will be measured using the Discourse Comprehension Test [42], which includes prerecorded stories with yes/no questions that assess story content related to explicit and implicit information, as well as main ideas and details. For discourse production, we will collect spoken discourse samples of various genres, including narratives, procedures, and expositions.

Narratives will be elicited in the following ways:

- Participants will be asked to generate 2 stories based on wordless picture sequences.
- Participants will be asked to talk about 2 personally relevant topics. Sample topics:
  “Tell me about something that happened to you recently or a recent event in your life that you feel comfortable sharing.”
  “Tell me about a challenge or problem you have encountered recently and how you addressed it.”

Procedural discourse will be elicited in the following ways:

- Participants will be asked to describe differences between 2 picture scenes that are seemingly identical and only differ in the presence/absence/substitution of some objects.
- Participants will be asked to describe plans for a hypothetical trip to another state, including what they will do to get ready to go, how they will travel, what they would bring, and what they would do at the destination [65].

Expository discourse will be elicited in the following way:

- Participants will be asked to explain their views on a topic. Sample topics:
  “Is technology helping people become smarter or making them less smart?”
  “Would you rather be all-knowing or all-powerful?”

Discourse samples will be audio- and/or video-recorded, transcribed, and segmented into T-units (ie, minimal terminal units), which are equivalent to sentences. T-unit segmentation will prepare the transcripts for further analysis. Transcripts will be analyzed for content (story completeness and global coherence) and organization (story grammar), which are critical dimensions of discourse ability sensitive to communication impairments in individuals with TBI. All discourse samples will be analyzed for global coherence. Only narratives will be analyzed for story completeness and story organization. We will also obtain word-level measures (eg, word counts, words per utterance). The story/discourse content and organization measures described below are not standardized assessments and, therefore, are limited in psychometric data. Reliability and correlations with other measures are presented, as available.

##### Story/Discourse Content

Story completeness will be evaluated by counting the number of critical components mentioned in the story using the protocol established by Lê et al [44], which is similar to other protocols for measuring content adequacy [66] and has demonstrated sensitivity to discourse impairments in TBI [13]. Global coherence will be evaluated using Wright et al’s [67] adaptation of Glosser and Deser’s [68] coherence measure. Each T-unit will be rated on a 4-point scale based on the extent to which the utterance reflects the overall theme of the discourse sample. Ratings will then be averaged across all T-units, providing an overall global coherence score.

##### Story Organization

For the story grammar measure, each discourse sample will be analyzed for the presence of episodes. An episode is operationalized as 3 main story grammar elements: (1) an initiating event that motivates a character’s action, (2) an attempt by the character to achieve the goal, and (3) a direct consequence of the character’s attempt. In the story grammar analysis, each T-unit will be evaluated for the presence of a main story grammar element. T-unit sequences with 3 story grammar elements are considered complete episodes, whereas those with 2 elements are considered incomplete. The primary story organization measure will be the proportion of T-units in episodes (complete or incomplete), which has demonstrated sensitivity to impaired discourse in TBI [69].

##### Story Goodness

Each discourse sample will be plotted using story content and story organization scores as coordinates according to the Story Goodness Index (SGI) protocol [44]. The SGI consists of 1 story content axis and 1 story organization axis. As there are 2 story content measures, 2 SGI plots will be constructed: (1) story completeness × story organization and (2) global coherence × story organization. The SGI allows for visualization of story goodness performance and categorization of story goodness ability into 4 groups (quadrants): (1) incomplete, organized stories; (2) complete, organized stories; (3) incomplete, disorganized stories; and (4) complete, disorganized stories. As the SGI uses the story content and organization measures described in the preceding sections, the reliability and correlational data for those measures apply to the SGI. The SGI has allowed for the identification of subgroups of discourse performance in previous studies [13,70].

##### Cognition

Cognition will be assessed as a potential moderator of preliminary intervention effects and to evaluate potential generalization. Working memory will be assessed using the Wechsler Adult Intelligence Scale—Fourth Edition Digit Span subtest [71]. Declarative memory will be assessed using the Wechsler Memory Scale—Fourth Edition Logical Memory I subtest [71]. Executive functions will be measured using the Repeatable Battery for the Assessment of Neuropsychological Status (Update) semantic fluency task [72] (eg, naming animals) and the Delis-Kaplan Executive Function System Tower Test [73]. Some cognitive tests may be audio- and/or video-recorded to facilitate analysis and scoring.

##### Functional Communication

Functional communication is “the strategic and effective employment of communication perception and production skills...to meet the individual’s participation goals within family, community, social, work, academic, and problem-solving contexts” [64] and will be assessed using the TBI-QOL COM short form [54]. It is a 9-item patient-reported outcome that uses a Likert-type response scale (eg, 1=cannot do to 5=none) and has an informant-report version. As an example, 1 item asks: “How much difficulty do you currently have speaking?” The TBI-QOL COM will be administered to participants and their informants. It will be administered via telephone to informants, who will be sent a postal copy of the TBI-QOL COM for reference.

##### Mental Health

As MH comorbidities may influence discourse, psychiatric symptom data, specifically related to PTSD, anxiety, and depression, will be collected. At this early stage of treatment development research, attempting to disentangle the unique contribution of MH factors and their potential impact on the discourse treatment being developed would be premature. However, the MH data collected will be an important preparatory step in evaluating the role of MH factors in a future, full-scale RCT of the treatment.

MH assessment will consist of 3 self-reported measures and will focus on the most common comorbidities of TBI: PTSD, anxiety, and depression. PTSD will be assessed using the PTSD Checklist for DSM-5 (20 items) [74]. Anxiety will be assessed using the 7-item Generalized Anxiety Disorder Scale [56]. Depression will be assessed using the 9-item Patient Health Questionnaire [57].

##### Pain and Headache

As pain and headache are common TBI comorbidities and may also influence discourse, we will collect self-reported information on the severity and functional impact of pain and headache. Pain will be assessed using the Pain, Enjoyment of Life and General Activity Scale (3 items) [75]. Headache will be assessed using the 6-item Headache Impact Test [59].

##### Daily Functioning

Functioning relative to daily activities and participation in life domains will be assessed using the MPAI-4 [61]. As this measure is already being administered at screening, it will be repeated only after training and at 1-month follow-up. Given that baseline testing will occur within approximately 1 month of screening, and that all participants will be beyond the acute stage of TBI—when most spontaneous recovery is expected to occur—MPAI-4 performance is expected to be stable during the period between screening and baseline. This approach will also reduce participant burden associated with readministration of the same assessment within a brief interval.

### Sample Size

Following the development of the initial version of the discourse treatment, 5-10 individuals will be recruited for the treatment development trial. The preliminary version of the treatment will be administered to this cohort for the primary purpose of “ironing out the kinks” in the protocol (eg, time spent introducing and practicing different treatment components, criteria for advancing to new content, duration of study sessions). This sample size was determined to be reasonable to allow for ongoing refinements to the treatment protocol as feedback is obtained from successive participants and is consistent with sample sizes used in other such treatment development trials [76].

In determining sample size for the phase 2 treatment feasibility trial, we followed guidelines by Leon et al [77] and Rounsaville et al [78], who recommend that a power analysis be used only for hypothesis-testing studies employing inferential statistics and advise against its use for treatment feasibility studies. Rather, Leon et al [77] recommend that the sample size for stage 1b treatment development research be based on the “pragmatics of recruitment and the necessities for examining feasibility.” Rounsaville et al [78] suggest 15-30 participants per cell. With a recruitment goal of 40 participants randomized to 1 of 2 treatment conditions and anticipated attrition of up to 25%, this would still leave 15 participants in each treatment arm, which is in line with treatment feasibility study recommendations according to guidelines by Leon et al [77] and Rounsaville et al [78].

### Statistical Analyses

#### Analysis of Feasibility Data

As this is a treatment development and feasibility study, the primary hypotheses concern the tolerability and acceptability of the treatment. To this end, we will calculate rates of treatment attendance and study retention. Analysis of tolerability will be performed by calculating the number of participants who (1) attend treatment sessions, (2) withdraw from treatment, (3) complete each of the assessment batteries, and (4) withdraw from assessment. The number of training and assessment sessions attended will also be calculated. Treatment dropouts, who only participate in pretraining assessments, will be compared with those who engage in the intervention on demographic, neurocognitive, and psychosocial variables. Treatment acceptability will be assessed by averaging participant satisfaction ratings for the treatment and assessment methods and participant feedback ratings regarding training content. Preliminary recruitment and participant flow data will be collected by calculating the number of participants who (1) call to inquire about the study, (2) qualify based on the brief telephone screening and sign informed consent, (3) qualify following intake screening, and (4) are randomized. Exit interviews will be analyzed qualitatively using a thematic analysis approach to provide an understanding of the impact of communication difficulties on the individual and participant feedback on various aspects of the treatment.

#### Descriptive Statistics

Descriptive statistics, including means and SDs for quantitative variables (including discourse, cognitive, MH, functional communication, and daily functioning variables in the assessment battery) and frequencies and percentages for qualitative variables, will be calculated for participant demographic variables, including age, education, and race, as well as ratings related to participant characteristics. We will also examine the data for outliers.

#### Statistical Analyses of Preliminary Treatment Effects

Although hypothesis testing is not the primary focus because this is a treatment feasibility study, we will perform several statistical analyses to provide preliminary estimates of effect sizes, score ranges, and variability (eg, means, SDs, measures of dispersion). For measures in the assessment battery, scatterplots will be constructed to allow visualization of performance patterns at each assessment time point. Measures of dispersion and distribution will also be obtained. Correlations will be calculated between the 2 discourse measures (story content and story organization) cross-sectionally, as well as between the discourse measures and other measures in the assessment battery. Associations between demographic and outcome variables (eg, discourse, cognition, MH, and functional measures) will be assessed via correlation and cross-tabulations. To compare treatment groups and estimate effect sizes, linear mixed models analyses will be performed on changes in outcomes from T1 (baseline) to T2 (after treatment) and from T2 to T3 (1-month follow-up). Linear mixed models are robust statistical analyses that account for missing data. In summary, these analyses will provide information regarding the feasibility of the discourse treatment, as well as inform our conceptual model of the relationships among the outcome variables.

### Ethical Considerations

#### IRB Approval

This project received initial approval from the Human Subjects Subcommittee (HSS), the institutional review board of VACHS, on July 2, 2021 (IRBNet ID 1628797; HSS # KL0001). All protocol amendments will be submitted to the VACHS HSS for approval before implementation. Where applicable, revisions will be made to consent forms, and participants will be reconsented as necessary.

#### Informed Consent

Informed consent will be obtained from all participants by study personnel in a private office. Potential participants will read the consent document (or have it read to them) and will be asked whether they have any questions about the study or the study procedures. Those who wish to be recontacted for a future study may check a box on the consent form indicating this.

#### Participant Compensation

Study participants will be paid as follows:

- Phase 1: US $40 for study screening; US $10 for each treatment session attended (16-20 sessions total); US $10 for homework assignments returned each week (approximately 8-12 total); US $25 for the posttreatment exit interview; and US $20 for posttreatment questionnaires.
- Phase 2: US $25 for study screening; US $10 for each treatment session attended (approximately 14-18 sessions total); US $5 for homework assignments returned each week (approximately 8-10 total); US $50 for each assessment battery (3 total); US $10 for the posttreatment exit interview; and US $10 for posttreatment questionnaires.

#### Privacy and Confidentiality

Privacy will be protected by conducting all study procedures in private offices. The PI will supervise and monitor all assessments and interventions. Identifying information will be protected by storing patient identifiers separately from other study data, coding patient data, and storing all information in locked offices or on a secure, access-restricted server.

Data will be collected during assessments and the study intervention. Data with no protected health information or personally identifiable information will subsequently be entered into electronic VA REDCap (Research Electronic Data Capture; Vanderbilt University), with access limited to designated study personnel.

Data will be analyzed by group, and identifying information will be removed from all forms and will not be directly linked to the data, as participants will be identified only by an assigned research number to protect their confidentiality. Compliance with the Health Insurance Portability and Accountability Act and confidentiality guidelines will be maintained, and only qualified research staff will have access to the database or coding key.

Research data will be maintained in accordance with current VA guidelines. Deidentified data will be used by research personnel to address study hypotheses. Any publications resulting from the study will use only deidentified data. No protected health information or individually identifiable health information will be disclosed to a non-VA entity.

#### Risks

This is a minimal risk study. The risks associated with this study are related to privacy, confidentiality, and psychological factors. Identifying information and sensitive medical information (ie, psychiatric history, substance use history) will be collected in this study. There is a potential risk to privacy and confidentiality if someone else becomes aware of the Veteran’s study participation; this will be communicated to participants in the consent form. Participants may experience psychological changes as they become more aware of their communication difficulties. Participants may become tired or frustrated while participating in the intervention or assessment battery. Participants will be offered breaks as needed and will always have the option of withdrawing from study participation.

In the unlikely event that a participant experiences a significant increase in psychiatric symptomatology (eg, exacerbation of PTSD symptoms), the consent form will specify that the participant’s MH provider will be contacted and that the participant may be hospitalized, even if this is not the participant’s wish.

Attribution of risk categories will be defined by the PI, with input from the coauthors, and will include grading by severity and relation to the study (definite, probable, possible, unlikely, or unrelated).

#### Monitoring

A data monitoring committee was not indicated for this study, as this is a minimal-risk, nonpharmacological study. The PI will oversee all study procedures and monitor the trial at least weekly during laboratory meetings.

#### Data Sharing

Final data sets underlying all publications resulting from the research may be shared approximately 6 months after publication. Deidentified data sets may be shared with other investigators, upon written request, under a data use agreement prohibiting the recipient from identifying or reidentifying any individual whose data are included in the data set. The data sharing agreement will also restrict redistribution to third parties and stipulate proper acknowledgment of the data source. These data sets will be shared in accordance with VA guidelines.

## Results

This study was funded in October 2021. Recruitment for phase 1 began on September 26, 2022. Phase 1 was completed with a total enrollment of 7 participants on November 17, 2023. Recruitment for phase 2 began on November 27, 2023. As of February 2026, phase 2 has been completed with a total enrollment of 34 participants. Data collection concluded on November 3, 2025. Scoring of measures has been concurrent with data collection and is ongoing. Scoring and data analysis are expected to continue through early-mid 2026. Results are expected to be published in peer-reviewed journals in late 2026 and early 2027. Findings will also be submitted to conferences for presentation, as well as reported in the trial registry.

## Discussion

### Anticipated Findings

The goal of this study is to advance cognitive-communicative intervention for people with TBI by developing an evidence- and theory-based narrative discourse treatment in the context of social communication that addresses communication breakdowns in both story content and organization. The study aims to develop a new manualized discourse treatment and collect preliminary tolerability, acceptability, and recruitment feasibility data.

The proposed research builds upon the contributions of a handful of prior discourse treatment studies and advances this work in several novel ways. This will be the first discourse treatment study to specifically target Veterans with TBI and the first manualized discourse-oriented treatment for TBI. No discourse treatment to date has been manualized into a standardized protocol that provides a step-by-step approach to treatment delivery. Second, the discourse treatment addresses both story content and organization and will be theory-based. Third, we will incorporate functional communication training (training of discourse skills for communication in participants’ everyday environments) to support carryover and generalization, which has been a missing ingredient in prior treatment attempts. Lastly, we will use a larger sample size than has been used to date.

Although an “active” control for the comparison group was considered, we chose to include a TAU-type control, as it would be premature to compare the proposed discourse treatment with other interventions at this early stage of treatment development. The TAU condition will allow us to pilot a control group that we would plan to use in any subsequent fully powered efficacy trial and to increase the likelihood of detecting a therapeutic effect.

### Conclusions

Discourse impairments are a hallmark of TBI and disrupt functioning across multiple life domains. Breakdowns in communication at the discourse level have a functional impact on persons with TBI, disrupting return to work, reintegration into communities, socialization, and quality of life. Veterans with TBI may experience even greater functional impairment due to physical and MH comorbidities. Improving discourse-level communication in a social context and through a structured approach would address a significant treatment gap for individuals with TBI.

The proposed discourse intervention aims to bridge this gap by targeting everyday storytelling embedded within the individual’s social context. Results from the proposed study will be used to further refine the treatment and assessment procedures based on participant feedback. If the study indicates that the treatment is tolerable and acceptable and that methods are feasible, the next step would be to design and implement a fully powered RCT of the discourse treatment, in which hypothesis testing would be appropriate, and efficacy data could be obtained. A full-scale RCT of the treatment would also provide the opportunity to explore the role of MH comorbidities, such as PTSD, anxiety, and depression, as potential moderators of discourse ability.

## Appendix

Multimedia Appendix 1 SPIRIT (Standard Protocol Items: Recommendations for Interventional Trials) checklist.

Multimedia Appendix 2 Structured trial summary with the World Health Organization (WHO) trial registration dataset.

## Abbreviations

CONSORT: Consolidated Standards of Reporting Trials
HSS: Human Subjects Subcommittee
LCFS: Rancho Los Amigos Level of Cognitive Functioning Scale—Revised
MH: mental health
MPAI-4: 4-item Mayo-Portland Adaptability Inventory
PI: principal investigator
PTSD: posttraumatic stress disorder
RCT: randomized controlled trial
REDCap: Research Electronic Data Capture
SBF: Structure Building Framework
SGI: Story Goodness Index
SMV: Service Members and Veterans
SPIRIT: Standard Protocol Items: Recommendations for Interventional Trials
STAR: setting, trigger, action, and result
TALE: Talking About Life Experiences
TAU: treatment as usual
TBI: traumatic brain injury
TBI-QOL COM: TBI Quality of Life Communication Item Bank
VACHS: VA Connecticut Healthcare System
WAB-R: Western Aphasia Battery—Revised
