# Supplementary material for: Talking About Life Experiences: Protocol for Treatment Development and a Feasibility Trial of a Novel Narrative Discourse Intervention for Individuals With Traumatic Brain Injury
Source: JMIR Res Protoc. 2026 Apr 24;15:e86329. doi: 10.2196/86329 (PMC13156538; doi:10.2196/86329)
Supplement: Multimedia Appendix 2 [file resprot_v15i1e86329_app2.pdf]

Supplemental Material 1: Structured trial summary with WHO Trial Registration Data Set

**WHO Data Set Item**	
Primary Registry and Trial Identifying Number	ClinicalTrials.gov ID NCT05008419
Date of Registration in Primary Registry	August 8, 2021
Secondary Identifying Numbers	C3494-W<br>1IK2RX003494-01A2 (U.S. VA Grant/Contract)
Sources of Monetary or Material Support	VA Office of Research and Development
Primary Sponsor	VA Office of Research and Development
Secondary Sponsor	N/A
Contact for Public Queries	Karen Lê, PhD<br>Principal Investigator<br>karen.le2@va.gov
Contact for Scientific Queries	Karen Lê, PhD<br>Principal Investigator<br>karen.le2@va.gov
Public Title	Narrative Discourse Treatment Development
Scientific Title	Remediating Narrative Discourse Impairments in Veterans with TBI: Initial Treatment Development
Countries of Recruitment	United States
Health Condition or Problems studied	Traumatic Brain Injury, Neurogenic Communication Disorders
Intervention	Behavioral: Narrative discourse treatment
Key Inclusion and Exclusion Criteria	Inclusion Criteria:<br>• Veteran with diagnosis of TBI with mild to moderate functional cognitive impairments

	• 18 years or older<br>• Self-report of communication difficulty following TBI that interferes with activities<br>• Can identify a significant other (e.g., spouse, family member, friend) who is able and willing to serve as an informant, who will verify discourse ability pre- and post-treatment<br>o The informant must be an individual with whom the participant engages in weekly social communication<br>• Adequate hearing and visual acuity to participate in study procedures<br>• Those who are willing and able to participate in telehealth sessions must have appropriate equipment and access, e.g., smartphone, tablet, computer with camera, internet access<br>• Stable housing<br>o Participants must have a safe, private and quiet environment in their home to engage in telehealth sessions<br>• English as a primary language<br><br>Exclusion Criteria:<br>• Penetrating head injury<br>• History of or current developmental disability (e.g., dyslexia), psychotic disorder, neurological illness, e.g., stroke, dementia, Parkinson's disease), aphasia or auditory processing disorder (APD)<br>• Current (past 30 days) diagnosis of alcohol or substance abuse<br>o An exception will be made for marijuana as a number of Veterans use marijuana occasionally to manage ailments, such as pain and PTSD<br>o Marijuana use must not occur regularly or interfere with daily functioning

	○ Inclusion of Veterans using marijuana aligns with the VA's position that Veterans will not be denied VA benefits because of marijuana use
Study Type	Purpose: Treatment<br><br>Allocation: Randomized controlled trial<br><br>Masking: Unblinded, no masking<br>Assignment: Parallel<br>Phase: Stage 1a and 1b
Date of First Enrollment	11/27/23
Sample Size	40
Recruitment Status	Active, not recruiting
Primary Outcomes	• Tolerability of treatment as measured by rates of attendance for all aspects of treatment [Time Frame: 2 months]<br>○ Rates of attendance for all aspects of treatment, calculated as the number of treatment sessions attended across all treatment phases divided by total number of treatment sessions.<br>• Feasibility of treatment as measured by dropout rate in the treatment condition [Time Frame: 2 months]<br>○ Dropout rate in the treatment condition will be calculated as the number of participants who do not complete treatment over total number of participants<br>• Acceptability of treatment as measured by a treatment satisfaction survey [Time Frame: 2 months]<br>○ Percentage of participants who rate satisfaction with the treatment based on Likert-type survey (e.g., endorse "satisfied" or "very satisfied")
Key Secondary Outcomes	None
Ethics Review	Status: Approved

	Approval date: 6/30/2021<br><br>Board: VA Connecticut Healthcare System Human Subjects Subcommittee
IPD Sharing Statement	Plan to Share IPD:<br><br>Yes<br><br>Plan Description:<br><br>Final data sets underlying all publications resulting from the research may be shared as per access criteria delineated below.<br><br>Time Frame:<br><br>6 months after publication<br><br>Access Criteria:<br><br>De-identified data sets may be shared with other investigators, upon written request, under a data use agreement prohibiting the recipient from identifying or re-identifying (or taking steps to identify or re-identify) any individual whose data is included in the dataset. The data sharing agreement will also restrict redistribution to third parties and proper acknowledgment of the data source. These data sets will be shared per guidelines provided by the VA Connecticut Healthcare System (VACHS) Information Security Officer.
